# Re-examining environmental correlates of *Plasmodium falciparum* malaria endemicity: a data-intensive variable selection approach

**DOI:** 10.1186/s12936-015-0574-x

**Published:** 2015-02-07

**Authors:** Daniel J Weiss, Bonnie Mappin, Ursula Dalrymple, Samir Bhatt, Ewan Cameron, Simon I Hay, Peter W Gething

**Affiliations:** Spatial Ecology and Epidemiology Group, Tinbergen Building, Department of Zoology, University of Oxford, Oxford, UK; Fogarty International Center, National Institutes of Health, Bethesda, MD USA

**Keywords:** *Plasmodium falciparum*, Malaria mapping, Africa

## Abstract

**Background:**

Malaria risk maps play an increasingly important role in disease control planning, implementation, and evaluation. The construction of these maps using modern geospatial techniques relies on covariate grids: continuous surfaces quantifying environmental factors that partially explain spatial heterogeneity in malaria endemicity. Although crucial, past variable selection processes for this purpose have often been subjective and *ad-hoc*, with many covariates used in modeling with little quantitative justification.

**Methods:**

This research consists of an extensive covariate construction and selection process for predicting *Plasmodium falciparum* parasite rates (*Pf*PR) in Africa for years 2000-2012. First, a literature review was conducted to establish a comprehensive list of covariates used for malaria mapping*.* Second, a library of covariate data was assembled to reflect this list, a process that included the construction of multiple, temporally dynamic datasets*.* Third, the resulting set of covariates was leveraged to create more than 50 million possible covariate terms via factorial combinations of different spatial and temporal aggregations, transformations, and pairwise interactions. Fourth, the expanded set of covariates was reduced via successive selection criteria to yield a robust covariate subset that was assessed using an out-of-sample validation approach.

**Results:**

The final covariate subset included predominately dynamic covariates and it substantially out-performed earlier sets used by the Malaria Atlas Project (MAP) for creating global malaria risk maps, with the pseudo-R^2^ value for the out-of-sample validation increasing from 0.43 to 0.52. Dynamic covariates improved the model, with 17 of the 20 new covariates consisting of monthly or annual products, but the selected covariates were typically interaction terms that included both dynamic and synoptic datasets. Thus the interplay between normal (i.e., long-term averages) and immediate conditions may be key for characterizing environmental controls on parasite rate.

**Conclusions:**

This analysis represents the first effort to systematically audit covariate utility for malaria mapping and then derive an objective, empirically based set of environmental covariates for modeling *Pf*PR. The new covariates produce more reliable representations of malaria risk patterns and how they are changing through time, and these covariates will be used to characterize spatially and temporally varying environmental conditions affecting *Pf*PR within a geostatistical-modeling framework, thus building upon previous research by MAP that produced global malaria maps for 2007 and 2010.

**Electronic supplementary material:**

The online version of this article (doi:10.1186/s12936-015-0574-x) contains supplementary material, which is available to authorized users.

## Background

Malaria risk maps play an increasingly important role in disease control planning, implementation, and evaluation, at scales ranging from local to global. Initial malaria mapping efforts resulted in manually drafted maps based largely on the expertise of a malariologist and the skill of an associated cartographer [1]. In more recent years, however, the production of malaria risk maps has been revolutionized through (i) the application of geographic information systems (GIS); (ii) the development of analytical techniques that extend statistical methodology into the spatial domain [2]; (iii) the evolution of spatial data products (i.e., covariates) that characterize natural and anthropogenic phenomena in a spatially explicit manner; and (iv) the proliferation of large-scale survey efforts that include measurements of malaria infection prevalence in geolocated communities [3]. Together these developments provide the tools and datasets necessary for producing malaria maps, including global products [4-6] that have a demonstrated utility within the malaria policy sphere [7].

Malaria mapping is an evolving research topic that is poised to expand in scope via studies that extend cross-sectional mapping methods into the temporal domain, thus allowing analysis of changing patterns of risk though time in an era of intensive control activity [8]. Increasing demand for more reliable and elaborate characterization of malaria risk patterns has fostered substantial innovation in spatiotemporal statistical methodology to improve predictive accuracy and provide robust characterizations of uncertainty [2,5,9,10]. Similarly, the quality, volume, and geographic coverage of data on malaria infection prevalence continue to increase, spearheaded by major international survey initiatives, such as the Demographic and Health Survey (DHS) program. In contrast to these advances, the covariate datasets selected for malaria mapping endeavors have received comparatively little concerted research attention. Although covariates often form the most important component of a geospatial malaria model when deriving predictions far from locations with observed data, the criteria for identifying and including particular covariates in a given study are often ill defined, with analysts relying on subjective *a priori* decisions about factors believed to be important, or simply selecting from a standard set of well-known variables based largely on their availability and convenience. Early examples of the use of environmental covariates in malaria risk mapping began appearing in the 1980s [11] and their inclusion in modern mapping efforts is now ubiquitous. Despite the increasing number of published malaria maps, a thorough meta-analysis of the spatial covariates utilized has not been undertaken*.*

In this context, the goals of this research were to (i) audit the universe of covariates that have been used in malaria risk maps; (ii) generate additional covariates where necessary, with a particular emphasis on the production of temporally dynamic datasets (i.e., covariates that vary through time); and (iii) develop an objective procedure to identify a robust covariate subset that will contribute the maximum predictive accuracy when incorporated in a spatio-temporal mapping exercise*.* This research fits into the broader research objectives of the Malaria Atlas Project (MAP) as it will (i) support spatio-temporal modeling of *Pf*PR in Africa and (ii) increase the accuracy of the *Pf*PR outputs for Africa, which in turn will improve results from downstream applications such as the modeling of clinical incidence and mortality. Furthermore, this research represents the due diligence necessary when transitioning from modeling only spatial heterogeneity in *Pf*PR for single time periods (i.e., the previous iterations of the MAP global products [4,5]) to producing outputs that characterize *Pf*PR through space and time.

## Methods

### Literature review of covariates used in malaria risk mapping

The initial step in this research was to conduct a comprehensive review of peer-reviewed publications to define and quantify the covariates that have been used in past malaria mapping endeavors*.* A Web of Knowledge search using the broad search terms ‘malaria’ and ‘map’ yielded 2,237 potential publications. These were screened manually and included only if they presented a map of malaria endemicity or risk that was generated using actual malaria infection response data or a related metric of transmission (such as entomological inoculation rate), and used at least one spatial covariate*.* To limit the scope of the analysis, the focus was only on the use of environmental covariates for mapping malaria, however such covariates have also been used as confounders in research designed to assess, for example, intervention exposures [12].

**Figure 1:**
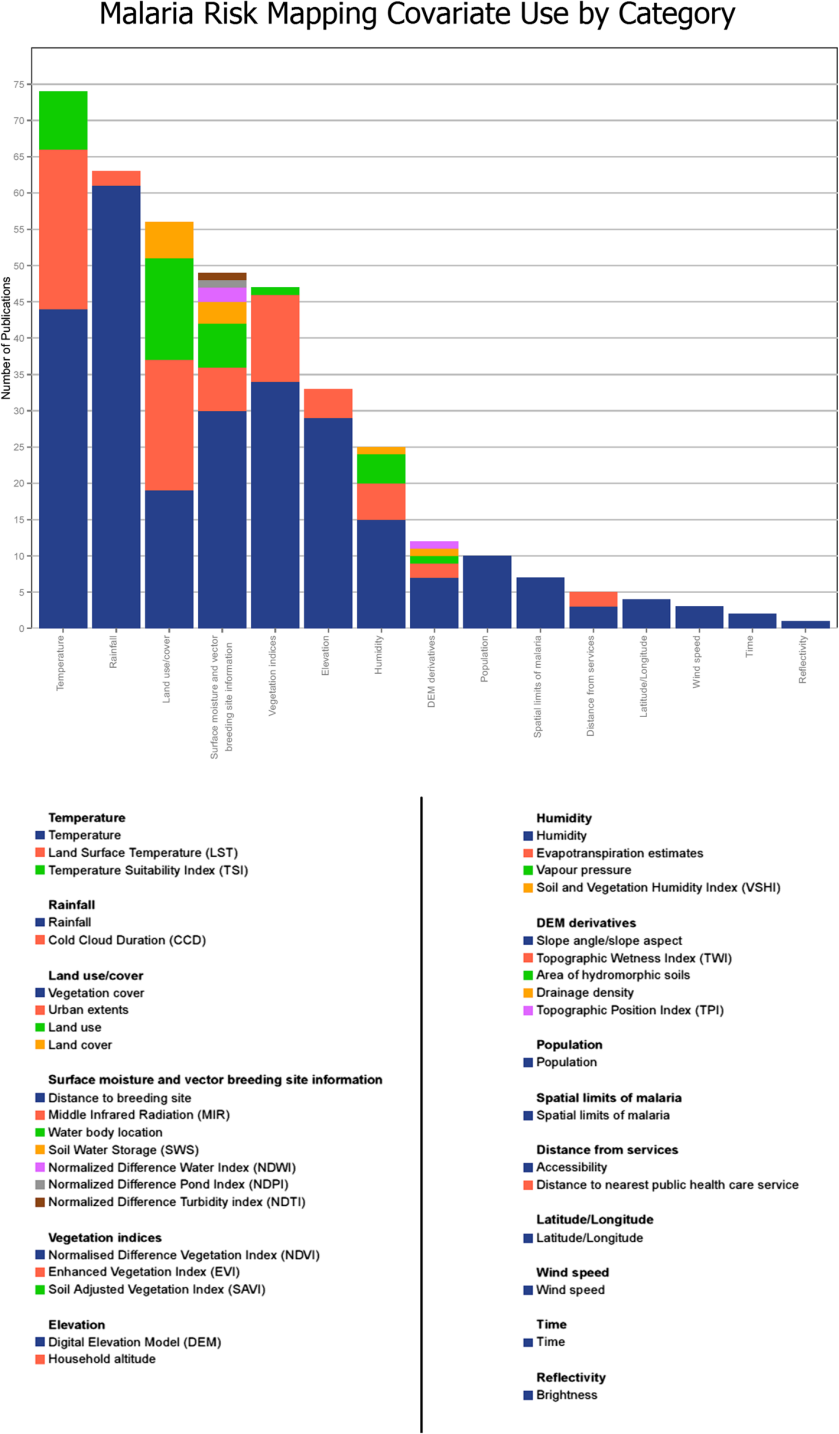
**Covariate use in malaria mapping.** Summarized results from research from 113 published studies.

From the resulting 113 studies (see Additional file 1), all covariates included in modeling were identified and summarized, and details such as the spatial resolution and underlying source of each covariate were recorded. The final map provided within each publication was also categorized by study extent (i.e., multinational, national, or subnational) and by continent of focus*.* The covariates were then grouped into logical categories, based on the covariates used and reasons for their selection, and tabulated to produce Figure 1*.*

The results of the meta-analysis demonstrate that climatic datasets quantifying temperature and precipitation are the most commonly used covariates for malaria mapping (>50% of studies), with metrics characterizing landscape properties (e.g., land cover type, vegetation indices, and metrics related to surface moisture and/or mosquito breeding sites) also being used extensively (>40%). Other covariate categories used are (in order of prevalence) elevation, humidity, products derived from digital elevation models (DEM), spatial limits of malaria (i.e., geospatial masks), distance from services, spatial coordinates (latitude and longitude), wind speed, time, and land surface reflectivity.

In many respects the consistency in the covariates used across malaria mapping endeavors is to be expected, as both the development of new spatial data products and the widespread adoption of these products in the malaria research community are slow processes*.* In general, the meta-analysis showed that the spatial scale of the mapping endeavor was an important influence on the variables used as some covariates (e.g., detailed maps of mosquito breeding sites) are available only for fine-scale studies due to the high computational and/or labor cost required to derive them*.* In contrast, the coarse scale of other covariates (e.g., global climatic datasets) may render them effectively useless for fine-scale analyses if, for example, all the response points fall within just a few grid cells.

Among the previous malaria mapping projects, the one that placed the greatest scrutiny on covariates was the MAP global malaria endemicity map for 2010 [5]. This research did an extensive exploration of many environmental covariates and ultimately selected the final set of covariates via an exhaustive test that compared all possible 10 and 20 term models. This project was limited, however, by a lack of dynamic covariates, no testing for non-linear relationships with malaria response data, and no exploration of interaction terms. This paper aims to improve upon the earlier MAP variable selection approach, which will be used as the benchmark for judging the results of the research presented.

### *Plasmodium falciparum* infection prevalence data

The geographic region, scale of analysis, time period, and parasite selected for this study were, respectively, Africa, 5 × 5 km pixels, 2000-2012, and *Plasmodium falciparum.* Response data consisted of observations of *P. falciparum* infection prevalence (termed parasite rate, abbreviated hereafter to *Pf*PR), each describing the number of individuals tested and found positive within a defined cluster represented geographically by a latitude and longitude coordinate attributed to each cluster centroid. The geographic coordinates for the cluster centroids were spatially offset in most cases, but the typical spatial offset (e.g., DHS offsets points by up to 5 km in rural areas) relative to the 5 × 5 km pixel size was such that cluster points would fall at most one pixel away from their correct location. Due to this limited offset of the points within the context of the raster analysis framework, no correction factor was applied to the cluster locations for this analysis. Each survey cluster is also associated with a survey date that allows the *Pf*PR to be temporally linked to dynamic covariates. These survey dates present two known limitations for this research: (i) the surveys often span multiple months and thus there is some uncertainty associated with the samples’ dates, and (2) positive diagnoses of malaria identified via the survey are not necessarily linked with the timing of infection, as the initial malaria infections may have occurred months or even years prior to the survey date.

**Figure 2:**
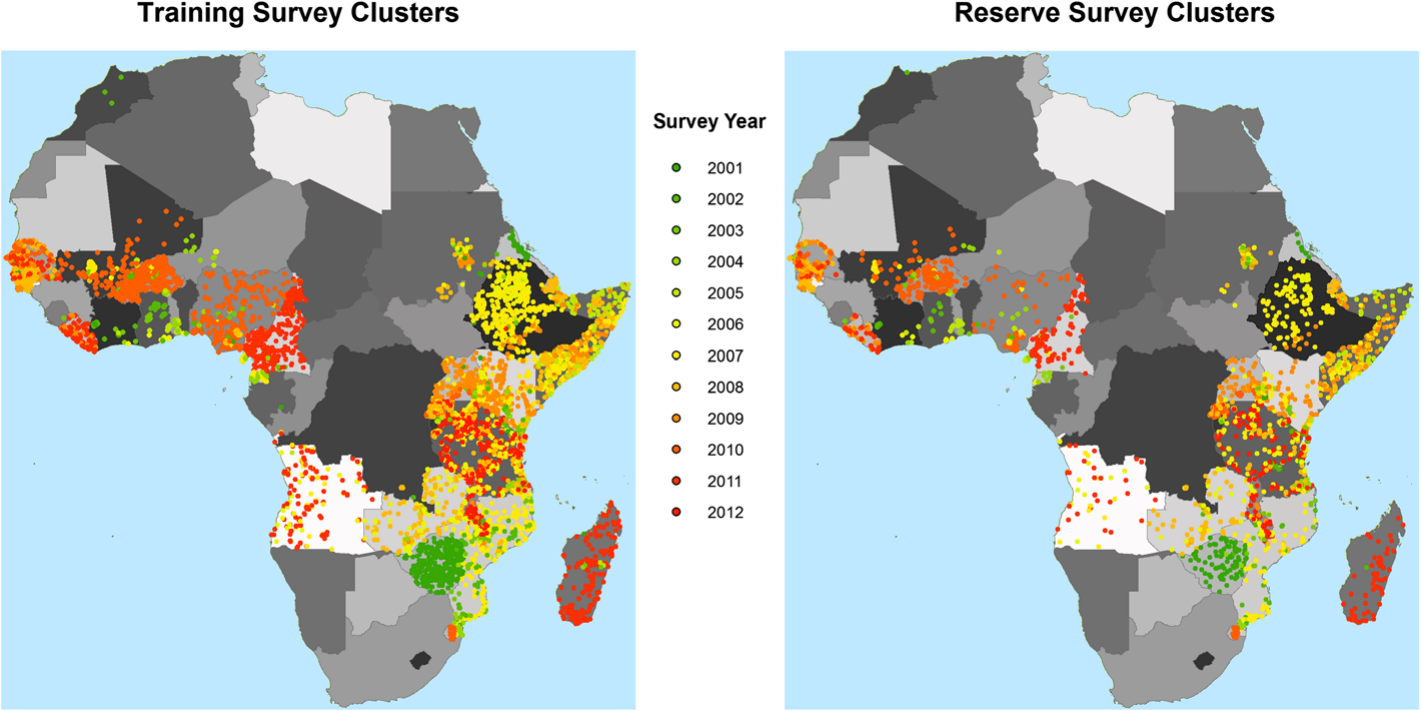
**Map of household clusters by year.** These survey points constitute the dependent variable for this research, with the training points used to parameterize the model, and the reserve points used for out-of-sample model validation.

The initial dataset consisted of 16,289 for years 2000-2012 [3], of which 10,903 were derived using microscopy, 5160 were derived using RDT, 122 were from RDT and confirmed by microscopy, and 104 were from RDT and confirmed by PCR. The data sources for the data are as follows: 5313 from DHS, 3195 from published literature, 1619 from other household surveys, 5900 from personal communication, and 262 from other sources. The majority of clusters (n = 9231) contained individuals above five years old, which necessitated an age adjustment to standardize the ages to years 2 through 10, which was done following Smith et al. [13]. The full set of points was trimmed to 16,253 clusters by removing all clusters with errors and/or an n of zero (i.e., all clusters with one or more individuals were used in this analysis), and then further reduced to 13,680 when points with matching spatial and temporal information (i.e., points falling within the same grid cell and collected during the same month and year) were aggregated*.* For validation purposes, 3,000 of 13,680 points were selected at random and reserved for out-of-sample validation, leaving a training dataset of 10,680 points*.* Figure 2 illustrates the distribution of survey points across space and time*.*

### Assembled covariate datasets

The datasets selected for this research were chosen to span the categories identified in the background literature review and to test the utility of including dynamic variables capable of characterizing inter- and intra-annual change in *Pf*PR*.* Key considerations when selecting variables were the spatial and temporal resolutions and extents of the datasets, which will be synchronized to the timing of the response variable collection time(s), thus supporting a robust spatio-temporal modeling approach*.* Wherever possible, dynamic datasets were used as the direct or proximate sources for the covariates*.* Such dynamic datasets represented a potentially important shift from synoptic datasets (i.e., single layers representing mean annual conditions) to covariates that accounted for changing landscape conditions through time (e.g., monthly, seasonal, or annual products)*.* As well as addressing change, the inclusion of dynamic datasets presented new opportunities that dramatically increased the range of potential predictor variables that could be derived by assessing, for example, multiple temporal lags, spatial and temporal summarization approaches, and deviation from normal conditions (i.e., temporal anomalies)*.* However, in some instances (e.g., elevation) this was obviously unnecessary, while other covariates (e.g., precipitation – see below) lacked the requisite data quality and/or fidelity at the spatial and temporal resolutions required in this study. In such cases, synoptic datasets were used instead. For dynamic covariates, values attributed to the malaria survey points were linked in both space and time, and included all desired lagged dates preceding the survey period*.*

### Temperature

The importance of temperature on mosquito survival, breeding, and biting rates has been well established [14,15] and, accordingly, temperature-related covariates are the most commonly used predictor variables in malaria mapping research*.* Spatio-temporal temperature patterns can be characterized in different ways and using different products*.* For this research four different temperature metrics were selected, each of which was derived from MODIS land surface temperature (LST) data [16] that were first gap-filled to remove missing values [17] and then aggregated to a monthly temporal resolution*.* The resulting datasets were (i) daytime LST; (ii) night-time LST; (iii) delta LST (i.e., daytime LST minus night-time LST); and (iv) temperature suitability for *P. falciparum* transmission [18]*.* Using remotely sensed data for characterizing temperature is advantageous as the LST values are directly attributable to each grid cell*.* As such, even in cases where an empirical model was used to fill a missing cell value, LST values are more representative of an *in situ* measurement than modeled datasets derived using measurements collected at widely dispersed meteorological base stations.

### Precipitation

Precipitation is associated with malaria risk directly, as it affects the availability of standing water utilized by mosquito larvae, and indirectly, by its influence in determining habitat types*.* Unlike temperature there are no readily available, remotely sensed precipitation datasets with the spatial and temporal resolutions and extents required for this analysis*.* As such, the global precipitation dataset produced by the WorldClim project [19] was used, after summarizing this dataset using Fourier analysis to create grids quantifying four amplitude metrics (the first of which is the mean precipitation value) and three phase metrics [20]*.* The resulting Fourier metrics characterized aspects of precipitation including the magnitude, variability, and seasonal rate of change for each grid cell*.* By summarizing many years of data into just a few, synoptic products, problematic aspects associated with modeling a contiguous raster dataset from sparsely distributed precipitation measurement stations were reduced*.* The downside of the Fourier summarization approach, however, was that long-term spatio-temporal trends (e.g., desertification) and other between-year variations were not retained in these datasets.

### Land cover

Land cover datasets characterize the spatial distribution and arrangement of land cover types, which impact malaria endemicity because vector mosquito species occupy distinct ecological niches (e.g., *Anopheles* species prefer dense forest cover)*.* Land cover datasets are typically produced by classifying remotely sensed imagery to create categorical outputs, wherein each pre-defined land cover type is codified using a single value*.* The categorical nature of land cover data can add complexity to a modeling process, but by acquiring land cover data of a higher spatial resolution than the analysis resolution, the categorical land cover can be converted into a set of continuous covariates, with one raster produced for each land cover class for each time period*.* For this analysis the International Geosphere-Biosphere Programme (IGBP) land cover classification available within the MODIS MCD12Q1 dataset [21] was utilized as it provided a dynamic (annual) land cover product for 2001-2012, with 17 classes (Table 1), and a 500-m spatial resolution*.* The categorical datasets were then summarized to produce 204 bands that quantified, for example, the fractional cover of evergreen broadleaf forest within a 5 × 5 km cell for 2005*.* To further test the utility of the land cover datasets, an additional metric was created that quantified the percentage of like adjacencies within the higher spatial resolution cells on a per-class basis, which is useful for characterizing landscape structure (i.e., whether the 500 × 500 m cells of a given class present within the 5 × 5 km output cell are contiguous or disaggregated).

**Table 1:** **IGBP land cover classes**

**Class number**	**IGBP class**
0	Water
1	Evergreen needleleaf forest
2	Evergreen broadleaf forest
3	Deciduous needleleaf forest
4	Deciduous broadleaf forest
5	Mixed forest
6	Closed shrublands
7	Open shrublands
8	Woody savannas
9	Savannas
10	Grasslands
11	Permanent wetlands
12	Croplands
13	Urban and built-up
14	Cropland/Natural vegetation mosaic
15	Snow and ice
16	Barren or sparsely vegetated

The International Geosphere-Biosphere Programme classification system utilized within the MODIS land cover product.

### Surface moisture and vector breeding site information

Information characterizing the availability of standing water is useful for assessing vector habitat quality as it can differentiate comparatively wet and dry grid cells*.* Candidate raster products for characterizing soil moisture include DEM derivatives (see below) and remotely sensed metrics designed to capture the moisture present in pixels*.* Examples of the latter include the normalized difference wetness index (NDWI) [22] and the tasseled cap wetness (TCW) approach [23] that has seen been adapted for use with MODIS bidirectional reflectance distribution function (BRDF) reflectance 16-Day composite imagery (MCD43B4.A2) [24]*.* TCW was selected for this analysis as previous research indicated it has a stronger association with surface water present within a pixel than NDWI [25]*.* Tasseled cap brightness (TCB) was also calculated for use as a covariate pertaining to moisture, as this metric will be high in areas with bare soils and/or abundant senescent vegetation that may be associated with dry seasonal phases*.* As with other MODIS products used in this analysis, TCW and TCB are dynamic products aggregated to a monthly temporal resolution and subsequently tested using multiple temporal lags*.* Lastly, the WorldClim potential evapotranspiration (PET) and aridity index (AI) synoptic products [26] were included to provide additional moisture-related covariates based, at least partially, on precipitation data.

### Vegetation indices

The density of vegetation cover is important for malaria as a means of characterizing vector habitat quality given the association between *Anopheles* mosquitoes and forested landscapes [27]*.* Vegetation indices are also potentially useful proxy datasets for precipitation, as datasets characterizing vegetation vigor may be indicative of the presence of standing water required for mosquito larval development sites*.* Furthermore, vegetation indices may be indicative of the topographic redistribution of water (e.g., hilltops are dry in comparison to valley bottoms) and thus further linked to standing water present in the landscape. However, the relationship between vegetation and available surface water is likely to be temporally asynchronous as precipitation (and potentially standing water) may precede the greening-up of vegetation as seasons transition from dry to wet*.* Likewise, vegetation may remain green for a period after seasonal precipitation ends and ephemeral surface water bodies have evaporated*.* As such, it is important to consider temporal lags when using vegetation cover as a potential proxy for moisture, and doing so requires datasets that capture intra-annual vegetation dynamics*.* Such vegetation dynamics are measurable using indices derived from multi-spectral remotely sensed imagery collected at regular intervals*.* For this research the enhanced vegetation index (EVI) [28] was utilized to characterize vegetation dynamics at a monthly temporal resolution for 2000-2012*.* As with the LST, TCW, and TCB products, the EVI dataset was produced using MODIS imagery that was gap-filled using the Weiss *et al*. [17] algorithm.

### Elevation

Elevation is used in malaria mapping primarily due to its association with temperature and precipitation, and this variable is expected to add little additional information to this analysis relative to high-quality climatic metrics*.* In the interest of thoroughness, however, elevation was included in this via an Africa-wide DEM created from the Shuttle Radar Topography Mission (SRTM) dataset [29], which had a native 90 m spatial resolution prior to spatial summarization processing that produced 5 × 5 km resolution products.

### DEM derivatives

High spatial resolution DEMs can be used to model the topographically controlled redistribution of water, thereby quantifying the relative moisture of each cell given uniform precipitation*.* Specific variables useful in this context include (i) slope angle, as water drains from steep slopes more quickly than from gradual slopes; (ii) flow accumulation, which sums the number of upslope grid cells that ultimately flow through cells located downslope and along flow paths; and (iii) topographic wetness index (TWI) [30] that combines slope and flow accumulation into a single metric*.* For this research the SRTM elevation dataset was converted into slope angle, flow accumulation, TWI, and six slope angle threshold datasets that quantify the fraction of the landscape with a slope less than one degree, two degrees, three degrees, and so on*.*

### Humidity

Humidity data were not directly included in this research as gridded humidity products are not available at the requisite spatial and temporal resolution necessary for the analysis*.* However, EVI, TCW, and delta LST are all potentially useful as proxies for humidity*.* EVI is related to humidity as vegetation density and vigor (i.e., absorption of photosynthetically active radiation) affect humidity due to their association with transpiration*.* TCW affects humidity as wet areas lose moisture via evaporation more readily than dry ones (at comparable temperatures)*.* Diurnal differences in temperature are indicative of humidity as atmospheric moisture insulates, retains heat, and heat is released as humidity condenses into dew, thus reducing diurnal temperature fluctuation.

### Socio-economic

Several additional variables were included in this analysis to account for anthropogenic factors associated with malaria transmission*.* As the relationship between these variables and malaria risk has not been extensively tested, several variants of each were included for exploratory purposes*.* Population density surfaces were included to account for the decreased prevalence of malaria in densely populated areas that results from poor habitat conditions for *Anopheles* mosquitoes (e.g., cities with large fractions of impervious surfaces, sparse vegetation, and poor surface water quality [31]). Specific population density surfaces tested were the AfriPop [32] and the gridded population of the world (GPW) [33] datasets. The AfriPop dataset was available at five-year intervals from 2000 to 2015, and to more thoroughly assess the relationship between *Pf*PR and population within this analysis, the dataset was converted into an annual product using a per-cell linear interpolation. Night-time lights data were included as a covariate due to the relationship between this variable and infrastructural development (e.g., electrification) [34], which is also related to urbanized areas likely to have lower *Pf*PR infection rates*.* Accessibility to cities with populations of more than 50,000 [35] was included as remote areas are less likely to have ready access to medical care useful for reducing the endemic *P. falciparum* infection level*.* This accessibility dataset consists of a cost-distance raster (i.e., a friction surface) derived by combining available transportation infrastructure datasets (e.g., road networks and airport locations) with nominal rates of travel using each mode of transport.

### Leveraging covariates

**Figure 3:**
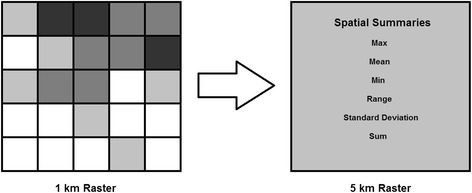
**Spatial summarization conceptual model.** Multiple finer-resolution raster cells are summarized to create a variety outputs that characterize intra-cell properties at the coarser-resolution.

To increase the utility of the covariates, and to avoid *a priori* assumptions about how the datasets are likely to relate to *Pf*PR, multiple techniques were applied to the covariate datasets to create many variants of each*.* The first of these techniques was spatial summarization. All of the covariates had native spatial resolutions that were higher than the 5 × 5 km analysis resolution and, as such, descriptive statistics (mean, minimum, maximum, range, standard deviation, and sum) (Figure 3) were calculated when multiple cells were aggregated to 5 × 5 km resolution. This procedure effectively increased the number of covariates by a factor of up to six*.* As previously mentioned, the land cover and slope threshold metrics were summarized using an approach that converted the categorical data into fractional cover (i.e., the proportion of the finer resolution cells meeting the classification criteria).

**Figure 4:**
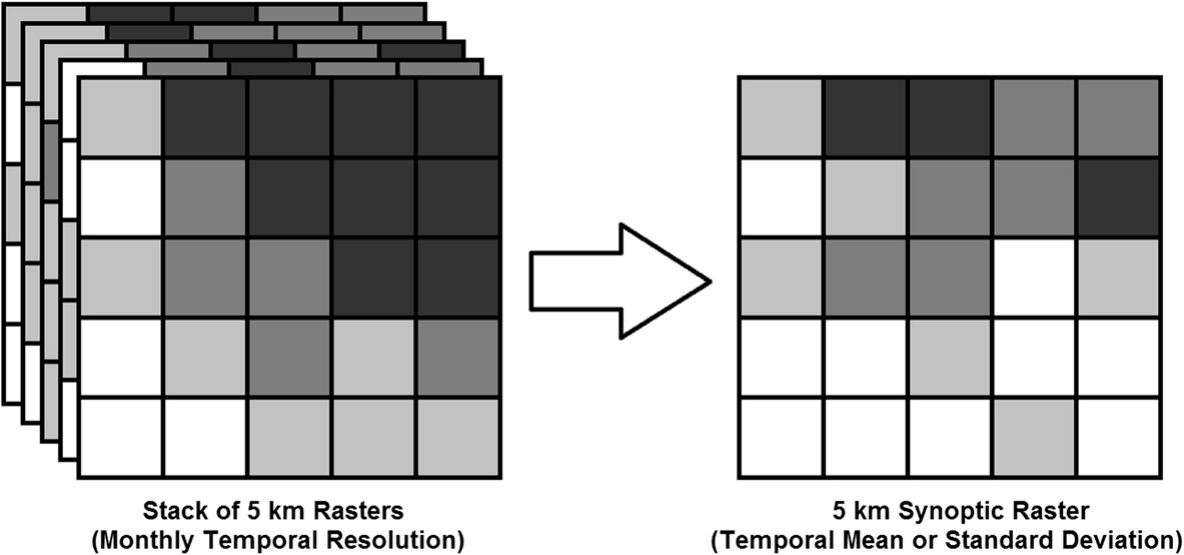
**Temporal summarization conceptual overview.** Summary statistics are derived from multiple rasters that are used directly as covariates and indirectly in the production of the anomaly covariates.

The dynamic nature of many of the covariates also provided the opportunity to summarize the data temporally, thus producing synoptic products (i.e., temporal means and standard deviations) using all dates in the covariate time-series and at both annual and monthly (e.g., the average conditions for all available months of March) time steps (Figure 4)*.* Note that, unlike the spatial summarization approach, minimum, maximum, and range were not calculated temporally since the monthly datasets were already the products of averaging and/or compositing and were thus unlikely to retain meaningful extreme values*.* The creation of synoptic products then enabled the derivation of anomaly products that characterized how different cell values in dynamic datasets were relative to synoptic values*.* Lastly, to account for environmental processes that have a lagged relationship with *Pf*PR, multiple versions of the dynamic products (i.e., LST, TSI, TCW, TCB, and EVI) were associated with each response point at monthly lags ranging from one to four months*.* Together the spatial and temporal summarization procedures, the creation of anomaly covariates, and the use of temporal lags increased the 33 base covariates to 922 variables associated with each response point (Table 2)*.* The covariates were linked to each survey point by extracting values from raster datasets based at the spatial location of the cluster and on the date of the survey (in cases where the survey spanned multiple months, the first month was used as the reference date). This procedure resulted in a merged data array, and it was at this stage that the table was split into the training and reserve datasets described above.

**Table 2:** **The initial set of covariates**

**Variable**	**Dynamic**	**Synoptic**	**Single**	**Spatial summary**	**Source**	**Total variants**
EVI	Monthly	Monthly	2000-2012	1 km to 5 km	MODIS derivative	137
LST day	Monthly	Monthly	2000-2012	1 km to 5 km	MODIS derivative	125
LST night	Monthly	Monthly	2000-2012	1 km to 5 km	MODIS derivative	125
LST delta	Monthly	Monthly	2000-2012	1 km to 5 km	MODIS derivative	52
TSI	Monthly	Monthly	2000-2012	1 km to 5 km	Modeled [18]	125
TCW	Monthly	Monthly	2000-2012	1 km to 5 km	MODIS derivative	125
TCB	Monthly	Monthly	2000-2012	1 km to 5 km	MODIS derivative	125
Slope angle			Static	90 m to 5 km	SRTM derivative	6
IGBP landcover	Annual			500 m to 5 km	MODIS product [21]	17
IGBP landcover pattern	Annual			500 m to 5 km	MODIS derivative	17
Flow accumulation			Static	90 m to 5 km	SRTM derivative	6
Slope thresholds			Static	90 m to 5 km	SRTM derivative	6
Elevation			Static	90 m to 5 km	SRTM [29]	6
TWI			Static	90 m to 5 km	SRTM derivative	6
AfriPop population	Annual			1 km to 5 km	AfriPop [32]	1
Accessibility			Static	1 km to 5 km	Nelson [35]	6
WorldClim AI			1950-2000	1 km to 5 km	WorldClim [26]	6
WorldClim PET			1950-2000	1 km to 5 km	WorldClim [26]	6
Nighttime lights			2012	1 km to 5 km	VIIRS	6
GRUMP			2010	1 km to 5 km	GRUMP 2010 [33]	6
GPW			2010	1 km to 5 km	GRUMP 2010 [33]	6
WorldClim preciptation			1950-2000	1 km to 5 km	WorldClim [19]	7

Basic details of the untransformed covariates that were associated with each survey point for the final model.

The next step in the covariate leveraging process was the consideration of potential non-linear relationships between *Pf*PR and the covariate datasets*.* As such, ten transformations (Table 3) were selected to capture a range of potential functional forms linking *Pf*PR to the covariates*.* Due to the use of log and square-root transforms, some variables were rescaled prior to transformation to eliminate values less than or equal to zero*.* The use of transformations increased the set of covariates by 11-fold (i.e., the untransformed variable and ten transformed versions), thereby establishing an effective covariate set of 10,142*.*

**Table 3:** **Transformations applied to the covariates**

**Name**	**Equation**
1) Untransformed	X* = *X*
2) Normalize	${X}^{*} = \left(X-\overline{X}\right)/{X}_{\sigma }$
3) Reciprocal	X* = 1/*X*
4) Log Base 10	${X}^{*} = \mathsf{l}\mathsf{o}{\mathsf{g}}_{\mathsf{10}}X$
5) Natural Log	X* = log_e_ *X*
6) Inverse Hyperbolic Sine (IHS)^a^	X* = log_e_(*X* + (*X* ^2^ + 1)^0.5^
7) Square	X* = *X* ^2^
8) Square Root	X* = *X* ^0.5^
9) Cube Root	X* = *X* ^*1*/*3*^
10) BoxCox Power Transformation	X* = *X* ^*λ*^
11) Absolute Normal	${X}^{*} = \left(X-\overline{X}\right)/{X}_{\sigma }$

Where *X* is a vector of values for a given variable, *X* ^*^ is the resulting transformed vector, $\overline{X}$ is the mean of *X*, *X*_*σ*_ is the standard deviation of *X* , and *λ* is the optimal univariate lambda for the BoxCox transformation calculated form the profile likelihood function in a precursor step.

^a^If the minimum value in the original data was zero, IHS was applied to the unadjusted values. If the minimum value was negative IHS was applied to the rescaled values.

**Figure 5:**
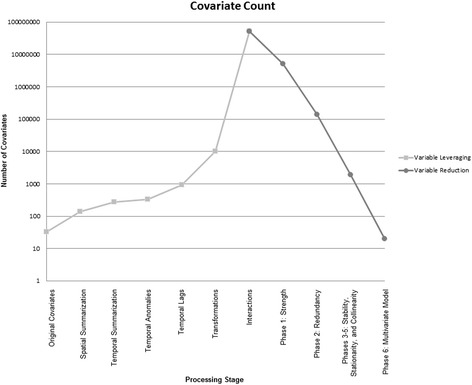
**The number of covariates relative to the phase of analysis.** The light grey points represent increasing covariates resulting from leveraging procedures, while the dark grey points represent the reduction in covariates by processing phase.

In real-world settings, each biotic and abiotic factor influencing transmission acts not alone, but rather in concert with all other such factors at a given location and time. To capture these combinatorial effects in a statistical model requires the inclusion of interaction terms between individual covariates. As such, all possible interactions from the set of 10,142 transformed covariates were considered, which produced a final covariate set in excess of 50 million possible variables (Figure 5).

### Variable selection

The statistical technique underlying this research is the binomial generalized linear model (GLM), with a dependent variable consisting of positive and negative *P. falciparum* counts tabulated for each survey cluster, and the independent variables consisting of the covariates described above*.* While the covariate leveraging process dramatically increased the number of covariates, the remaining steps were designed to reduce the number of the covariates and ultimately produce a robust multivariate model of computationally manageable size using an objective and rational approach*.* All of the following phases were applied only to the training dataset, with the reserve (i.e., holdout) dataset retained for out-of-sample validation of the final model*.* Each successive selection phase was configured to reduce the set of covariates by approximately an order of magnitude (Figure 5)*.*

### Phase one: strength of relationship

Phase one consisted of calculating GLM results for all 50 + million possible covariates (i.e., the bivariate GLM relationship between each environmental covariate and *Pf*PR) and then ranking (i.e., sorting) the output based on the Akaike Information Criteria (AIC, [36]) of the models*.* The resulting set of variables was then trimmed to retain only the best 10% of all models*.* Although the choice of a 10% threshold is somewhat arbitrary, it was guided by the rationale that poor predictor variables need not be retained with such a vast number of better predictors (i.e., more than five million) left to choose from*.* Furthermore, the remaining variables still retained multiple forms of all 33 base covariates alone or in combination with other variables via an interaction term.

### Phase two: reducing redundancy

Due to the use of transformations and the creation of interaction terms, multiple variants of the same base covariate are present within the set of approximately five million remaining variables*.* Because the variants (e.g., interaction terms consisting of the same two variables, but with different transforms applied to each) are highly correlated it was unnecessary to retain more than one per covariate*.* To remove the redundant variants, the list of covariates (sorted to prioritize binomial GLM models with the lowest AIC values) was thinned to retain only the best variant of each possible variable or interaction*.* This phase reduced the set of covariates to approximately 137,000*.*

### Phase three: stability

The remaining covariates were then subjected to a bootstrapping procedure whereby a binomial GLM was run for 1,000 random draws, each containing one-fifth of the training dataset survey points (i.e., 2,136 points in each draw)*.* An identical randomization seed was used when testing each covariate to ensure comparability of the results*.* The results of this phase were then ranked according to the consistency of the AIC parameter across all the draws, thus providing a means of characterizing which covariates were the most robust predictors of *Pf*PR across a wide range of potential subsets of survey clusters*.*

### Phase four: spatial stationarity

This phase is conceptually similar to the assessment of stability, but instead of taking random draws of the survey clusters, the clusters were first grouped in subsets based on their spatial location*.* Four separate spatial zonal schemes were selected for this purpose, three of which were simple grid-based approaches that used square regions across Africa of widths two, four, and eight decimal degrees (using WGS-1984 geographic coordinates)*.* The fourth zonal scheme utilized was The Nature Conservancy ecoregion dataset [37] that categorizes areas based on their biophysical and ecological properties*.* For each of the four zonal datasets a binomial GLM was run using all available points within each sub-region, and covariates were tracked for their statistical significance across all sub-regional models*.* The resulting tallies of significance across all possible zones were then used to rank the covariates based on their spatial stationarity*.* Unlike the analysis of stability, the stationarity rank is also inherently a test of spatial scale, as the highest ranked predictors were those that were significantly related to *Pf*PR variability at a variety of scales.

### Phase five: collinearity

The stability and stationarity ranks from the preceding two phases were then combined to produce a composite rank intended to preferentially select variables that were the most consistent predictors of *Pf*PR. The remaining covariates were sorted using this composite rank, and the resulting data table was thinned to remove highly collinear variables (using a threshold of absolute correlations in excess of 0.70 [38])*.* This process yielded a thinned set of 1,887 covariates. The role of endogenous relationships was not considered beyond this test for collinearity, as the ultimate (downstream) goal of this research was to establish the best predictive PR map for Africa rather than assessing the causal inference for individual variables. Furthermore, given the scope of the dataset, approaches suitable for assessing endogenous relationships (e.g., regularization or the application of structural equations) were computationally impractical.

### Phase six: multivariate analysis

The final stage in the analysis was to produce a multivariate model of manageable size that was comparable to the model used by MAP to create the 2010 global *Pf*PR map [5], which had 20 covariate terms*.* An adapted version of the probabilistic variable selection approach [39] was used for this purpose, as an exhaustive search for 20 term models from a set of 1,887 covariates was computationally prohibitive*.* This probabilistic variable selection approach is predicated on selecting (initially) random models and iteratively modifying the probability of each term being included in the next quasi-random draw based on that term’s impact upon previous models*.* While the model that emerges from the probabilistic variable selection procedure is not necessarily the optimum model, this approach will produce a model that approaches the maximum predictive capacity of the covariate dataset.

### Evaluation of final covariate set performance

The final covariate model was tested for in- and out-of-sample predictive accuracy using a variety of test metrics. For comparison, the same tests were performed using the 20-covariate set from the earlier MAP study [5]. The metrics used were (i) the AIC; (ii) root mean squared error (RMSE); (iii) the squared correlation between the counts of actual and predicted positive *P. falciparum* individuals at each cluster; (iv) the squared correlation between the measured and modeled *Pf*PR; and (v) the pseudo *R*^2^ value [40]*.* Multiple metrics were used for comparing the multivariate models as no single metric alone is ideal for the task*.* AIC is imperfect as it is only applicable to the training dataset and it is a relative measure, so it is challenging to assess the degree of improvement*.* RMSE is convenient because this metric is in units of actual *Pf* positive individuals, but is also heavily influenced by clusters with large sample sizes where small errors in *Pf*PR equate to many incorrectly classified individuals, and thus disproportionally impacts the mean error for all clusters*.* The *R*^2^ count metric is also directly impacted by the number of individuals in the cluster, as large clusters will typically have more individuals testing positive and vice versa*.* In contrast, the *R*^2^ ratio metric ignores cluster size, which penalizes the multi-phase variable selection process that was optimized for binomial GLM models*.* In other words, the model is designed to be the best predictor for all individuals in all clusters while the *R*^2^ for the ratios effectively weights all clusters equally, ignoring the fact that the model will perform better in the larger clusters*.* The pseudo *R*^2^ is a compromise metric that overcomes the shortcomings of the other metrics (i.e., it takes clusters size into account effectively), and produces a value analogous to conventional *R*^2^, however this metric is somewhat atypical within epidemiological research. Lastly, to evaluate the relative performance of the full 20-covariate model *versus* those with smaller numbers of terms, the same suite of test metrics was computed for the best-performing model of size 1,2,..20 covariates, for both the new covariate suite and that from the earlier MAP study [5].

## Results

The 20 terms selected in the final multivariate model are listed in Table 4*.* All terms in this final model consisted of interactions (i.e., the product of two covariates), which is not surprising given the additional information gleaned from combining variables*.* The 20 interaction terms were created using 32 of the 10,142 univariate terms that were evaluated, and several of these terms were used more than once. Among the 32 terms used (Table 5), 15 were metrics characterizing temperature, with daytime LST being used five times, night-time LST and delta LST both being used four times, and TSI being used twice. Among the other metrics, EVI was used four times, land cover classes and TCW were each used three times, slope thresholds and precipitation metrics were each used twice, and PET, elevation, and TCB were used once each.

**Table 4:** **The final set of malaria covariates**

**Variable**	**Coefficient**	**Std. Error**	**Z value**	**Term 1**	**Transform 1**	**Term 2**	**Transform 2**
(Intercept)	−5.83E + 00	6.33E-02	−92.1				
v1	−3.07E-03	4.12E-05	−74.36	T17	7	T23	11
v2	−5.72E-01	7.31E-03	−78.19	T13	11	T5	7
v3	6.42E-01	2.10E-02	30.54	T11	10	T32	7
v4	1.71E + 00	2.12E-02	80.87	T3	8	T22	4
v5	3.70E-01	1.17E-02	31.65	T3	11	T10	3
v6	−7.44E-01	6.12E-02	−12.16	T2	9	T10	10
v7	9.84E-01	1.51E-02	65.4	T1	11	T10	3
v8	−1.43E-01	4.77E-03	−29.96	T27	3	T18	2
v9	1.55E-04	2.37E-06	65.65	T8	7	T26	6
v10	−6.78E-01	1.19E-02	−57.05	T9	6	T7	10
v11	2.59E-01	4.76E-03	54.32	T31	2	T21	2
v12	−1.36E-03	3.87E-05	−35.21	T19	7	T24	11
v13	7.60E-01	2.27E-02	33.4	T3	7	T32	10
v14	3.04E + 00	9.74E-02	31.24	T14	3	T7	10
v15	−6.75E-02	2.49E-03	−27.08	T20	1	T25	3
v16	−5.32E-01	2.38E-02	−22.35	T16	10	T29	3
v17	−1.04E + 00	2.22E-02	−46.79	T15	4	T28	2
v18	−8.20E-03	1.86E-04	−44.13	T12	10	T28	10
v19	2.02E + 01	5.96E-01	34	T4	4	T11	10
v20	2.94E-01	2.07E-02	14.22	T30	10	T6	3

The covariates remaining in the multivariate model, along with their transforms, coefficients, and z-scores. Note that in cases where transform three (i.e., the reciprocal) was used the sign of the coefficient is typically the opposite of what would be expected*.* The terms are further explained in Table 5.

**Table 5:** **The initial covariates used in the final multivariate model**

**Term ID**	**Variable**	**Spatial summary**	**Temporal summary**	**Temporal anomaly**	**Temporal lag**	**Multiple use**
T1	EVI	Max	Dynamic monthly	Difference from synoptic annual	3 months	-
T2	EVI	Max	Dynamic monthly	-	4 months	-
T3	EVI	Mean	Dynamic monthly	Difference from synoptic annual	Varies	3
T4	EVI	Min	Dynamic monthly	Difference from synoptic monthly	0 months	-
T5	Land cover class 12 - Croplands	Fractional	Dynamic annual	-	-	-
T6	Land cover class 13 – Urban	Fractional	Dynamic annual	-	-	-
T7	Land cover class 14 – Cropland/Natural vegetation mosaic	Fractional	Dynamic annual	-	-	2
T8	LST day	Max	Synoptic monthly mean	-	0 months	-
T9	LST day	Mean	Synoptic monthly standard deviation	-	1 month	-
T10	LST day	Range	Synoptic Monthly Mean	-	Varies	3
T11	LST day	Standard deviation	Synoptic Monthly Mean	-	2 months	-
T12	LST Day	Standard deviation	Synoptic monthly standard deviation	-	3 months	-
T13	LST night	Mean	Synoptic annual mean	-	-	-
T14	LST night	Mean	Dynamic monthly	Difference from synoptic annual	0 months	-
T15	LST night	Min	Synoptic monthly standard deviation	-	4 months	-
T16	LST night	Range	Synoptic monthly mean	-	2 months	-
T17	LST diurnal change	Max	Synoptic annual mean	-	-	-
T18	LST diurnal change	Max	Dynamic monthly	-	0 months	-
T19	LST diurnal change	Mean	Dynamic monthly	-	0 months	-
T20	LST diurnal change	Mean	Synoptic monthly mean	-	1 month	-
T21	PET	Mean	Static	-	-	-
T22	Precipitation (A0)	Mean	Static	-	-	-
T23	Precipitation (A2)	Mean	Static	-	-	-
T24	Slope less than 2 degrees	Fractional	Static	-	-	-
T25	Slope less than 5 degrees	Fractional	Static	-	-	-
T26	Elevation	Max	Static	-	-	-
T27	TCB	Range	Synoptic monthly mean	-	0 months	-
T28	TCW	Min	Dynamic monthly	-	Varies	2
T29	TCW	Range	Synoptic monthly mean	-	2 months	2
T30	TCW	Standard deviation	Dynamic monthly	-	3 months	-
T31	TSI	Mean	Synoptic monthly standard Deviation	-	2 months	-
T32	TSI	Min	Dynamic monthly	-	3 months	-

Each was utilized as one half of the interaction terms shown in Table 4.

Of the final 20 terms, 14 were fully dynamic, with each month and year combination having a unique raster layer, thus resulting in over 150 layers per covariate. Four terms were dynamic at the monthly scale only, meaning 12 raster layers constitute each covariate (e.g., all survey clusters collected in January being associated with a single layer). One term was dynamic at the annual scale, with one layer for each of the 13 years in the temporal extent of this research. Lastly, just one term was strictly synoptic and consisted of a single layer linked to all survey clusters. Despite the prevalence of dynamic covariates in the final multivariate model, only one was the product of two fully dynamic covariates (i.e., only one of the 20 final terms was formed as the interaction between two covariates that each consisted of unique layers for each month of all years).

**Figure 6:**
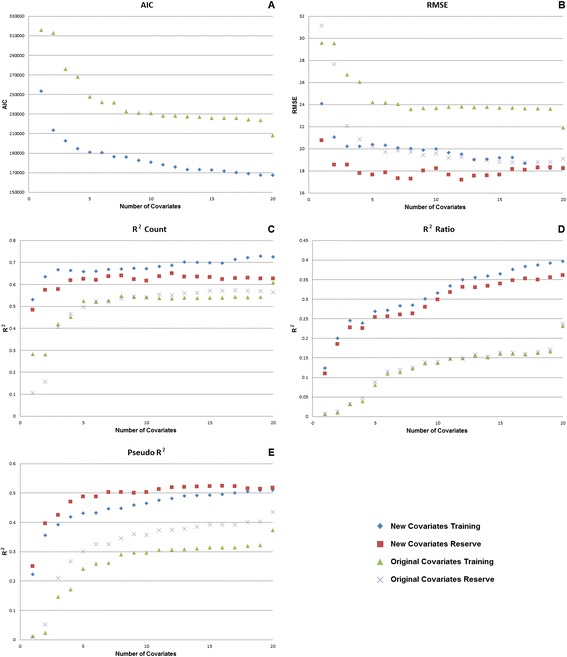
**Comparisons of model performance when using the old vs new covariate sets.** The panels **A-E** depict five different test metrics to illustrate the model improvement relative to the number of terms in the model.

Table 6 contains a direct comparison between the covariate dataset used to create the MAP 2010 global *Pf*PR map [5]*.* The new covariates out-perform the old set according to all of the test metrics selected to evaluate the multivariate models. As expected, the results derived using the training data are generally superior to those achieved from the reserve dataset, but the similarity in results from these models suggest that neither the old or new covariates are overfit. Exploring the variables by sequentially adding them to the model further demonstrates the utility of the new covariates, as the first three new covariates alone are more effective at predicting *Pf*PR than the full set of 20 older covariates (Figure 6) used to create the 2010 map*.*

**Table 6:** **Covariate comparison for modeling** ***Pf*** **PR in Africa**

**Dataset**	**Metric**	**New**	**Old**
Training	AIC	167553.4	208208.9
Training	RMSE	18.315	21.928
Training	R^2^(count)	0.7251	0.6088
Training	R^2^(ratio)	0.3970	0.2309
Training	Pseudo R^2^	0.5102	0.3739
*Reserve*	*RMSE*	*18.228*	*19.097*
*Reserve*	*R* ^*2*^ *(count)*	*0.6266*	*0.5608*
*Reserve*	*R* ^*2*^ *(ratio)*	*0.3633*	*0.2382*
*Reserve*	*Pseudo R* ^*2*^	*0.5174*	*0.4356*

The comparison between the newly created covariates and the covariates used to create the MAP 2010 global product. Statistics for the reserve dataset are shown in italics.

**Figure 7:**
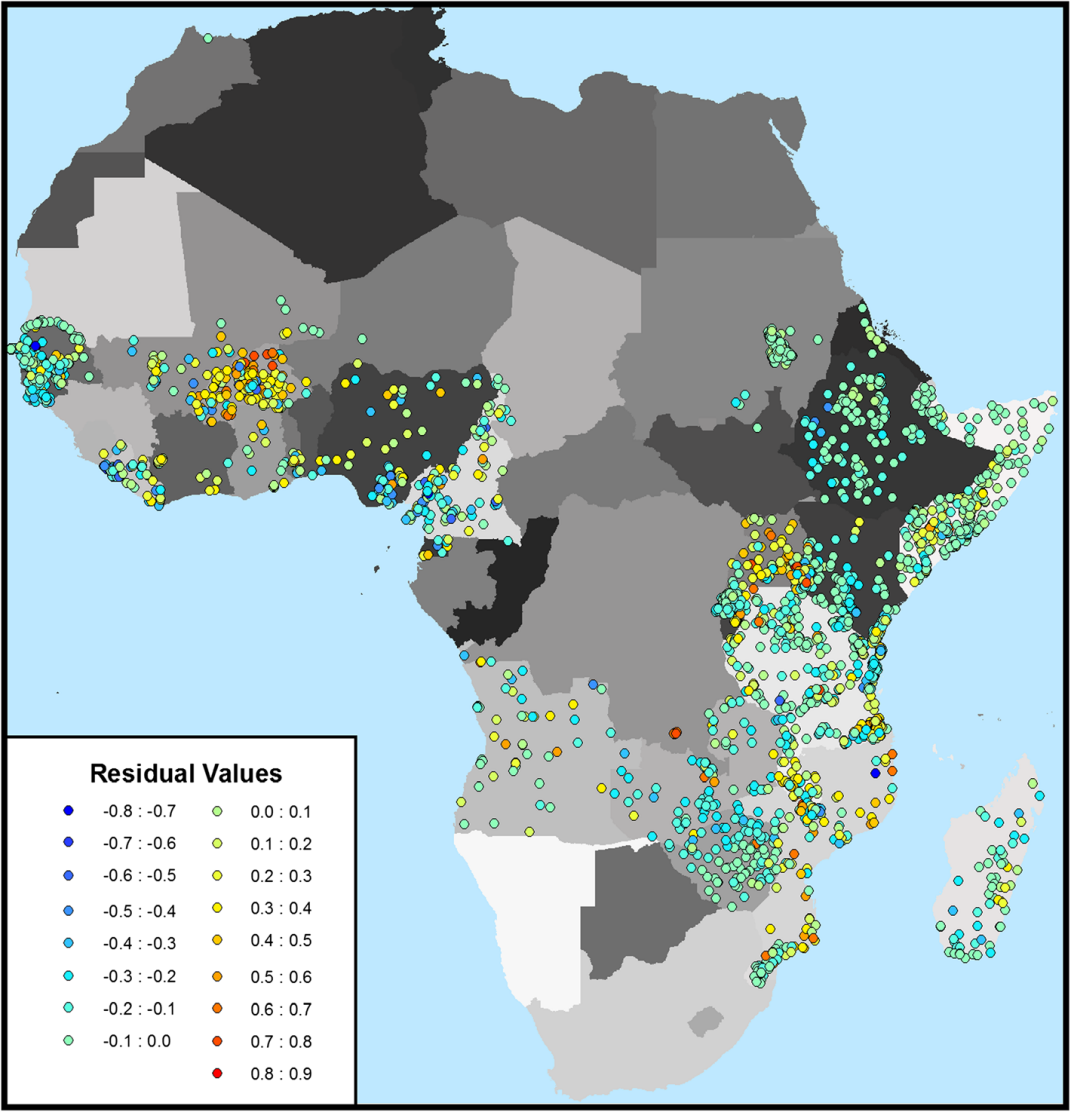
**Map of the** ***Pf*** **PR residual values associated with clusters within the reserve dataset.** The residuals were calculated for each reserve cluster (n = 3000) as the measured *Pf*PR minus the modeled *Pf*PR.

**Figure 8:**
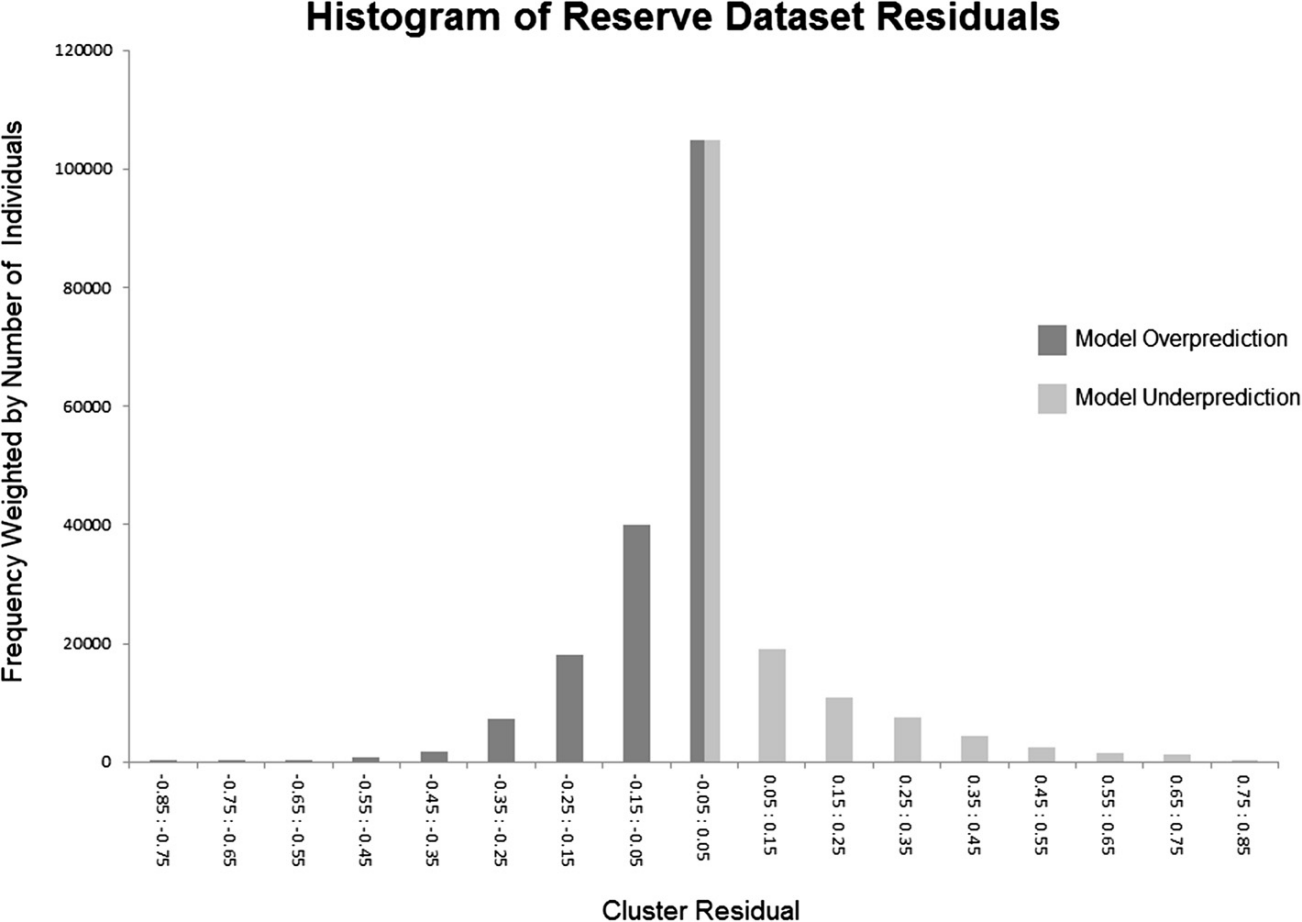
**The histogram of** ***Pf*** **PR residual values for clusters in the reserve dataset.** Each cluster point was weighted by the number of individuals residing in the cluster to reflect the results of a model based upon binomial GLMs.

The final model was further tested for potential systematic errors and geographic biases (Figures 7 and 8) using only the reserve dataset. The map of residuals suggests that the model slightly over predicts *Pf*PR for most of Africa, with the exception of Burkina Faso and Uganda where the model has a more pronounced under prediction. The histogram reveals that the model classifies *Pf*PR for clusters in which 74.6% the individuals in the reserve dataset (n = 219,751) reside within +/- 0.15. The model was then tested for potential issues related to the use of dynamic covariates, as these inherently contain temporal trends that function at various scales (e.g., inter-annually and seasonally). Thus it is conceivable that long-term trends in the covariates, if present, could be spuriously correlated with declines in malaria resulting from ongoing intervention efforts, which have expanded substantially since 2005. Given the potential for such an issue to negatively impact downstream attempts to accurately attribute declines in *Pf*PR to specific intervention efforts, it was necessary to test for such trends at this stage. To do so the full temporal range of each of the 19 dynamic covariates was intersected with 1,000 random points distributed throughout the *Pf* endemic areas of Africa. The resulting dataset was then tested for temporal trends and it was found that all covariates had slopes near zero.

## Discussion

The variable selection approach developed in this research represents an objective, data-driven methodology that resulted in a set of covariates that are quantitatively superior for predictive modeling of *Pf*PR to those used in previous MAP malaria mapping endeavors*.* A drawback of this approach, however, is a decrease in model interpretability, as the final set of covariates is more challenging to interpret than previous, conceptually simpler, covariate sets*.* Model accuracy was prioritized over model interpretability in this research as MAP products are typically used as input layers by other researchers with little regard to the relative importance of individual covariates. As such, the focus of this research was on developing the best predictive model in lieu of one that is easier to interpret, to maximize the accuracy of the resulting *Pf*PR maps and therefore limit downstream modeling error. While the variable leveraging process makes the relative influence of single covariates on *Pf*PR more challenging to discern, it should be noted that the root datasets from which the covariates were derived are well established as useful predictors of the spatial distribution of malaria endemicity*.* As such, the methodology represents an approach for greatly increasing the predictive capacity of the final model without requiring the acquisition and use of additional, and potentially costly, covariate datasets. It should also be noted that the methodology developed in this paper may have utility for other research endeavors, but the full set of the covariate leveraging and reduction phases need not be applied in all analyses. Using a subset of the covariate leveraging and/or reducing steps, in particular the exclusion of interaction terms, is also likely to increase the interpretability of the results.

While this research was not designed for assessing single covariates within the final model, it is possible to investigate whether the covariates, as used in the model, make sense biologically. For example, the covariate ‘V4’ is the variable with the highest absolute z-score (i.e., +80.87) found the final model. This variable is an interaction term comprised of (i) mean annual precipitation and (ii) the difference between monthly EVI and the 13-year average EVI value (i.e., a monthly anomaly variable that reflects seasonally changing vegetation vigor). Each term was transformed prior to deriving their product, with a log transform applied to the precipitation variable and a square root transformation applied to the EVI anomaly variable (after adding the minimum value of the raw EVI anomaly term to all values in the dataset since a real root cannot be derived for a negative number). No lag is associated with the EVI anomaly dataset for this covariate. Within the multivariate model the resulting interaction term (i.e., ‘V4’) has a positive coefficient, indicating that *Pf*PR is positively correlated with the combination of annual precipitation and higher than normal EVI. The biological interpretation of this variable is that places with above average EVI are likely indicating seasonal peaks in precipitation that cannot be gleaned from the synoptic precipitation layer (i.e., the monthly EVI anomaly produces a proxy for dynamic precipitation when linked with the synoptic precipitation dataset). Thus the positive coefficient associated with ‘V4’ makes sense as it indicates that, for areas generally moist enough for malaria, seasonal spikes in greenness are correlated with higher *Pf*PR.

A limitation of this research relates to the subsequent production of the raster datasets associated with the selected covariates*.* While the analysis presented was based on a simple data table, the actual creation of *Pf*PR maps first requires the production of covariate datasets that, in the case of fully dynamic variables, amount to over 150 layers per covariate*.* The production time and data storage requirements associated with such variables were an important consideration when choosing to cap the number of covariates at 20*.* However, exploratory analysis of models with more terms supports this decision, as larger models did not substantially improve the ability to predict *Pf*PR in the reserve (i.e., validation) datasets*.* This finding occurred despite the fact that test metrics for in-sample validation of training models continued to improve with the addition of more variables (e.g., with a least angle regression approach the ‘optimized’ training model contained several hundred covariates)*.*

An important sub-question for this research relates to discerning the value of dynamic covariates in comparison to synoptic products*.* The composition of the final set of covariates, 95% of which were dynamic, suggests that malaria mapping benefits from the use of dynamic variables*.* However, the continued utility of synoptic variables, often in combination with dynamic variables as one half of an interaction term, suggests that using both dynamic and synoptic variables is the optimum approach for spatio-temporal malaria mapping*.* This finding is reasonable, as long-term synoptic patterns and short-term variability are both likely to be important drivers of *Pf*PR. Furthermore, this finding is potentially useful to other researchers operating with resource limitations that preclude such an ambitious variable selection process, as it illustrates the continuing utility of synoptic variables (i.e., those more readily available) in the absence of dynamic covariate sets.

The next stage in the malaria mapping process will be to incorporate the outcomes of this research in a spatio-temporal (Bayesian) geostatistical framework that will further increase the predictive capacity of the new covariates by taking advantage of residual spatial and temporal structure present within the response dataset*.* For example, the eventual application of this spatio-temporal model will correct for the spatial heterogeneity in the model residuals illustrated in Figure 7. The net result will be a spatio-temporal ‘cube’ of *Pf*PR whereby each pixel will have an estimated rate for each of the 13 years of the analysis. This dataset will then serve as a key input to downstream applications such as the derivation of incidence and mortality. While there is an argument that such a spatio-temporal model should have been used in the variable selection process presented here, the computational efficiency of the simpler, GLM-based approach enabled a much more ambitious exploration of the covariate space (i.e., over 50 million potential covariates examined) than would have been possible with a full spatio-temporal model*.* Furthermore, an ideal statistical model for malaria mapping would not require a spatio-temporal model at all, as the covariate datasets would fully account for the spatial heterogeneity evident in the response dataset, thus leaving no spatial or temporal structure to exploit*.* For this reason, basing the variable selection process on GLM models ensures that the influence of the covariates within the final geostatistical model is prioritized over spatial and temporal structure within the response dataset.

## Conclusions

The variable leveraging and selection approach described in this research produced a set of covariates with much more predictive capacity than the set utilized to create the 2010 MAP malaria map [5]*.* The improvement resulted from the utilization of spatial and temporal summarizations of dynamic covariates, which further enabled the production of covariates including synoptic covariates, covariates accounting for temporally lagged relationships, and covariates characterizing temporal anomalies (i.e., deviation from synoptic norms)*.* The methodology developed in this research has potential utility for researchers seeking to maximize the utility of a rich set of environmental covariates while also limiting subjective decisions within the variable selection process. The improved covariates stand to significantly improve the eventual *Pf*PR mapping results generated by more sophisticated spatio-temporal modeling approaches, and serve as a guide for researchers wishing to undertake malaria mapping at a variety of scales worldwide. Lastly, the selected set of covariates has been directly incorporated within the current round of spatial-temporal *Pf*PR modeling by MAP, the results of which were released in early 2015 as part of the WHO World Malaria Report.

## Additional file

Additional file 1:
**Supplementary information: the list of citations from the malaria mapping literature review.**
