# Supplementary material for: Re-examining environmental correlates of Plasmodium falciparum malaria endemicity: a data-intensive variable selection approach
Source: Malar J. 2015 Feb 7;14:68. doi: 10.1186/s12936-015-0574-x (PMC4333887; doi:10.1186/s12936-015-0574-x)
Supplement: Additional file 1: — Supplementary information: the list of citations from the malaria mapping literature review. [file 12936_2015_574_MOESM1_ESM.docx]

1. Ahmadian Marj a., Mobasheri MR, Valadan zoej MJ, Rezaei Y, Abaei MR: **Exploring the use of satellite images in the estimation of potential malaria outbreak regions**. *Environ Hazards* 2009, **8**:89–100.

2. Alegana V a, Atkinson PM, Wright J a, Kamwi R, Uusiku P, Katokele S, Snow RW, Noor AM: **Estimation of malaria incidence in northern Namibia in 2009 using Bayesian conditional-autoregressive spatial-temporal models.** *Spat Spatiotemporal Epidemiol* 2013, **7**:25–36.

3. Alemu K, Worku A, Berhane Y: **Malaria infection has spatial, temporal, and spatiotemporal heterogeneity in unstable malaria transmission areas in northwest Ethiopia.** *PLoS One* 2013, **8**:e79966.

4. Amek N, Bayoh N, Hamel M, Lindblade K a, Gimnig JE, Odhiambo F, Laserson KF, Slutsker L, Smith T, Vounatsou P: **Spatial and temporal dynamics of malaria transmission in rural Western Kenya.** *Parasit Vectors* 2012, **5**:86.

5. Ayala D, Costantini C, Ose K, Kamdem GC, Antonio-Nkondjio C, Agbor J-P, Awono-Ambene P, Fontenille D, Simard F: **Habitat suitability and ecological niche profile of major malaria vectors in Cameroon.** *Malar J* 2009, **8**:307.

6. Ayele DG, Zewotir TT, Mwambi HG: **Spatial distribution of malaria problem in three regions of Ethiopia.** *Malar J* 2013, **12**:207.

7. Baeza A, Bouma MJ, Dobson AP, Dhiman R, Srivastava HC, Pascual M: **Climate forcing and desert malaria: the effect of irrigation.** *Malar J* 2011, **10**:190.

8. Balls MJ, Bødker R, Thomas CJ, Kisinza W, Msangeni H a, Lindsay SW: **Effect of topography on the risk of malaria infection in the Usambara Mountains, Tanzania.** *Trans R Soc Trop Med Hyg* 2004, **98**:400–8.

9. Bennett A, Kazembe L, Mathanga DP, Kinyoki D, Ali D, Snow RW, Noor AM: **Mapping malaria transmission intensity in Malawi, 2000-2010.** *Am J Trop Med Hyg* 2013, **89**:840–9.

10. Bertrand Sudre, Massimiliano Rossi, Wim Van Bortel, Kostas Danis, Agoritsa Baka, Nikos Vakalis and JCS: **Mapping Environmental Suitability for Malaria Transmission, Greece**. *Emerg Infect Dis* 2013.

11. Bøgh C, Lindsay Sw, Clarke Se, Dean A, Jawara M, Pinder M, Thomas Cj: **High Spatial Resolution Mapping Of Malaria Transmission Risk In The Gambia, West Africa, Using Landsat^TM^ Satellite Imagery Claus**. *Am J Trop Med Hyg* 2007, **76**:875–881.

12. Cairns M, Roca-Feltrer A, Garske T, Wilson AL, Diallo D, Milligan PJ, Ghani AC, Greenwood BM: **Estimating the potential public health impact of seasonal malaria chemoprevention in African children.** *Nat Commun* 2012, **3**:881.

13. Caminade C, Ndione J a., Kebe CMF, Jones a. E, Danuor S, Tay S, Tourre YM, Lacaux J-P, Vignolles C, Duchemin JB, Jeanne I, Morse a. P: **Mapping Rift Valley fever and malaria risk over West Africa using climatic indicators**. *Atmos Sci Lett* 2011, **12**:96–103.

14. Ceccato P, Vancutsem C, Klaver R, Rowland J, Connor SJ: **A vectorial capacity product to monitor changing malaria transmission potential in epidemic regions of Africa.** *J Trop Med* 2012, **2012**:595948.

15. CH V, EM N, MR. D: **Use of remote sensing to study the influence of environmental changes on malaria distribution in the Brazilian Amazon**. *Cad Saude Publica* 2006, **22**:517–526.

16. Cohen JM, Dlamini S, Novotny JM, Kandula D, Kunene S, Tatem AJ: **Rapid case-based mapping of seasonal malaria transmission risk for strategic elimination planning in Swaziland.** *Malar J* 2013, **12**:61.

17. Craig MH, Sharp BL, Mabaso MLH, Kleinschmidt I: **Developing a spatial-statistical model and map of historical malaria prevalence in Botswana using a staged variable selection procedure.** *Int J Health Geogr* 2007, **6**:44.

18. Dambach P, Machault V, Lacaux J-P, Vignolles C, Sié A, Sauerborn R: **Utilization of combined remote sensing techniques to detect environmental variables influencing malaria vector densities in rural West Africa.** *Int J Health Geogr* 2012, **11**:8.

19. Dambach P, Sié A, Lacaux J-P, Vignolles C, Machault V, Sauerborn R: **Using high spatial resolution remote sensing for risk mapping of malaria occurrence in the Nouna district, Burkina Faso.** *Glob Health Action* 2009, **2**:1–7.

20. de Oliveira EC, dos Santos ES, Zeilhofer P, Souza-Santos R, Atanaka-Santos M: **Geographic information systems and logistic regression for high-resolution malaria risk mapping in a rural settlement of the southern Brazilian Amazon.** *Malar J* 2013, **12**:420.

21. Dogan HM, Cetin I, Egri M: **Spatiotemporal change and ecological modelling of malaria in Turkey by means of geographic information systems.** *Trans R Soc Trop Med Hyg* 2010, **104**:726–32.

22. Elyazar IRF, Gething PW, Patil AP, Rogayah H, Kusriastuti R, Wismarini DM, Tarmizi SN, Baird JK, Hay SI: **Plasmodium falciparum malaria endemicity in Indonesia in 2010.** *PLoS One* 2011, **6**:e21315.

23. Elyazar IRF, Gething PW, Patil AP, Rogayah H, Sariwati E, Palupi NW, Tarmizi SN, Kusriastuti R, Baird JK, Hay SI: **Plasmodium vivax malaria endemicity in Indonesia in 2010.** *PLoS One* 2012, **7**:e37325.

24. Garske T, Ferguson NM, Ghani AC: **Estimating Air Temperature and Its Influence on Malaria Transmission across Africa**. *PLoS One* 2013, **8**:e56487.

25. Gemperli a, Vounatsou P, Sogoba N, Smith T: **Malaria mapping using transmission models: application to survey data from Mali.** *Am J Epidemiol* 2006, **163**:289–97.

26. Gemperli A, Sogoba N, Fondjo E, Mabaso M, Bagayoko M, Briët OJT, Anderegg D, Liebe J, Smith T, Vounatsou P: **Mapping malaria transmission in West and Central Africa.** *Trop Med Int Health* 2006, **11**:1032–46.

27. Gething PW, Patil AP, Smith DL, Guerra C a, Elyazar IRF, Johnston GL, Tatem AJ, Hay SI: **A new world malaria map: Plasmodium falciparum endemicity in 2010.** *Malar J* 2011, **10**:378.

28. Gething PW, Van Boeckel TP, Smith DL, Guerra C a, Patil AP, Snow RW, Hay SI: **Modelling the global constraints of temperature on transmission of Plasmodium falciparum and P. vivax.** *Parasit Vectors* 2011, **4**:92.

29. Giardina F, Gosoniu L, Konate L, Diouf MB, Perry R, Gaye O, Faye O, Vounatsou P: **Estimating the Burden of Malaria in Senegal: Bayesian Zero-Inflated Binomial Geostatistical Modeling of the MIS 2008 Data**. *PLoS One* 2012, **7**:e32625.

30. Gosoniu L, Vounatsou P, Sogoba N, Maire N, Smith T: **Mapping malaria risk in West Africa using a Bayesian nonparametric non-stationary model**. *Comput Stat Data Anal* 2009, **53**:3358–3371.

31. Gosoniu L, Vounatsou P, Sogoba N, Smith T: **Bayesian modelling of geostatistical malaria risk data.** *Geospat Health* 2006, **1**:127–39.

32. Gosoniu L, Msengwa A, Lengeler C, Vounatsou P: **Spatially explicit burden estimates of malaria in Tanzania: bayesian geostatistical modeling of the malaria indicator survey data.** *PLoS One* 2012, **7**:e23966.

33. Gosoniu L, Veta AM, Vounatsou P: **Bayesian geostatistical modeling of Malaria Indicator Survey data in Angola.** *PLoS One* 2010, **5**:e9322.

34. Gosoniu L, Vounatsou P: **Non-stationary partition modeling of geostatistical data for malaria risk mapping**. *J Appl Stat* 2011, **38**:3–13.

35. Guerra C a, Snow RW, Hay SI: **Defining the global spatial limits of malaria transmission in 2005.** *Adv Parasitol* 2006, **62**:157–79.

36. Guerra C a, Gikandi PW, Tatem AJ, Noor AM, Smith DL, Hay SI, Snow RW: **The limits and intensity of Plasmodium falciparum transmission: implications for malaria control and elimination worldwide.** *PLoS Med* 2008, **5**:e38.

37. Guerra C a, Howes RE, Patil AP, Gething PW, Van Boeckel TP, Temperley WH, Kabaria CW, Tatem AJ, Manh BH, Elyazar IRF, Baird JK, Snow RW, Hay SI: **The international limits and population at risk of Plasmodium vivax transmission in 2009.** *PLoS Negl Trop Dis* 2010, **4**:e774.

38. Hanafi-Bojd a a, Vatandoost H, Oshaghi M a, Charrahy Z, Haghdoost a a, Zamani G, Abedi F, Sedaghat MM, Soltani M, Shahi M, Raeisi a: **Spatial analysis and mapping of malaria risk in an endemic area, south of Iran: a GIS based decision making for planning of control.** *Acta Trop* 2012, **122**:132–7.

39. Haque U, Magalhães RJS, Reid HL, Clements AC a, Ahmed SM, Islam A, Yamamoto T, Haque R, Glass GE: **Spatial prediction of malaria prevalence in an endemic area of Bangladesh.** *Malar J* 2010, **9**:120.

40. Hay SI, Guerra C a, Gething PW, Patil AP, Tatem AJ, Noor AM, Kabaria CW, Manh BH, Elyazar IRF, Brooker S, Smith DL, Moyeed R a, Snow RW: **A world malaria map: Plasmodium falciparum endemicity in 2007.** *PLoS Med* 2009, **6**:e1000048.

41. Hay SI, Okiro E a, Gething PW, Patil AP, Tatem AJ, Guerra C a, Snow RW: **Estimating the global clinical burden of Plasmodium falciparum malaria in 2007.** *PLoS Med* 2010, **7**:e1000290.

42. Howes RE, Piel FB, Patil AP, Nyangiri O a, Gething PW, Dewi M, Hogg MM, Battle KE, Padilla CD, Baird JK, Hay SI: **G6PD deficiency prevalence and estimates of affected populations in malaria endemic countries: a geostatistical model-based map.** *PLoS Med* 2012, **9**:e1001339.

43. Huang F, Zhou S, Zhang S, Zhang H, Li W: **Meteorological factors-based spatio-temporal mapping and predicting malaria in central China.** *Am J Trop Med Hyg* 2011, **85**:560–7.

44. Incardona S, Vong S, Chiv L, Lim P, Nhem S, Sem R, Khim N, Doung S, Mercereau-Puijalon O, Fandeur T: **Large-scale malaria survey in Cambodia: novel insights on species distribution and risk factors.** *Malar J* 2007, **6**:37.

45. Jury MR, Kanemba AD: **A climate-based model for malaria prediction in southeastern Africa**. *S Afr J Sci* 2007, **103**:57–62.

46. Kazembe LN: **Spatial modelling and risk factors of malaria incidence in northern Malawi.** *Acta Trop* 2007, **102**:126–37.

47. Kazembe LN, Kleinschmidt I, Holtz TH, Sharp BL: **Spatial analysis and mapping of malaria risk in Malawi using point-referenced prevalence of infection data.** *Int J Health Geogr* 2006, **5**:41.

48. Kleinschmidt I, Bagayoko M, Clarke GP, Craig M, Le Sueur D: **A spatial statistical approach to malaria mapping.** *Int J Epidemiol* 2000, **29**:355–61.

49. Kleinschmidt I, Omumbo J, Briët O, van de Giesen N, Sogoba N, Mensah NK, Windmeijer P, Moussa M, Teuscher T: **An empirical malaria distribution map for West Africa.** *Trop Med Int Health* 2001, **6**:779–86.

50. Kleinschmidt I, Sharp BL, Clarke GP, Curtis B, Fraser C: **Use of generalized linear mixed models in the spatial analysis of small-area malaria incidence rates in Kwazulu Natal, South Africa.** *Am J Epidemiol* 2001, **153**:1213–21.

51. Klinkenberg E, van der Hoek W, Amerasinghe FP: **A malaria risk analysis in an irrigated area in Sri Lanka**. *Acta Trop* 2004, **89**:215–225.

52. Kulkarni M a, Desrochers RE, Kerr JT: **High resolution niche models of malaria vectors in northern Tanzania: a new capacity to predict malaria risk?**. *PLoS One* 2010, **5**:e9396.

53. Lek-Uthai U, Sangsayan J, Kachenchart B, Kulpradit K, Sujirarat D, Honda K: **Novel ellipsoid spatial analysis for determining malaria risk at the village level.** *Acta Trop* 2010, **116**:51–60.

54. Leonardo LR, Rivera PT, Crisostomo BA, Sarol JN, Bantayan NC, Tiu WU BN: **A study of the environmental determinants of malaria and schistosomiasis in the Philippines using Remote Sensing and Geographic Information Systems**. *Parassitologica* 2005, **47**:105–114.

55. Lindsay SW, Hole DG, Hutchinson R a, Richards S a, Willis SG: **Assessing the future threat from vivax malaria in the United Kingdom using two markedly different modelling approaches.** *Malar J* 2010, **9**:70.

56. Liu J, Chen X: **Relationship of remote sensing normalized differential vegetation index to Anopheles density and malaria incidence rate.** *Biomed Environamental Sci* 2006, **19**:130–132.

57. Mabaso MLH, Craig M, Vounatsou P, Smith T: **Towards empirical description of malaria seasonality in southern Africa: the example of Zimbabwe.** *Trop Med Int Health* 2005, **10**:909–18.

58. Machault V, Vignolles C, Pagès F, Gadiaga L, Gaye A, Sokhna C, Trape J-F, Lacaux J-P, Rogier C: **Spatial heterogeneity and temporal evolution of malaria transmission risk in Dakar, Senegal, according to remotely sensed environmental data.** *Malar J* 2010, **9**:252.

59. Machault V, Vignolles C, Pagès F, Gadiaga L, Tourre YM, Gaye A, Sokhna C, Trape J-F, Lacaux J-P, Rogier C: **Risk mapping of Anopheles gambiae s.l. densities using remotely-sensed environmental and meteorological data in an urban area: Dakar, Senegal.** *PLoS One* 2012, **7**:e50674.

60. Magalhães RJS, Langa A, Pedro JM, Sousa- JC, Clements ACA, Nery SV: **Role of malnutrition and parasite infections in the spatial variation in children’ s anaemia risk in northern Angola**. *Geospat Health* 2013, **7**:341–354.

61. Magalhães RJS, Langa A, Sousa-Figueiredo JC, Clements AC a, Nery SV: **Finding malaria hot-spots in northern Angola: the role of individual, household and environmental factors within a meso-endemic area.** *Malar J* 2012, **11**:385.

62. Medina D, Bevilacqua M, Cárdenas L, Morales LG, Palis YR, Martínez A, Behm V, Moreno J, Magris M: **Risk map of malaria transmission in the Caura river basin , Venezuela.** *BOLETÍN Malariol Y SALUD Ambient* 2011, **L1**:129–144.

63. Messina JP, Taylor SM, Meshnick SR, Linke AM, Tshefu AK, Atua B, Mwandagalirwa K, Emch M: **Population, behavioural and environmental drivers of malaria prevalence in the Democratic Republic of Congo.** *Malar J* 2011, **10**:161.

64. Moffett A, Shackelford N, Sarkar S: **Malaria in Africa: vector species’ niche models and relative risk maps.** *PLoS One* 2007, **2**:e824.

65. Moiroux N, Bio-Bangana AS, Djènontin A, Chandre F, Corbel V, Guis H: **Modelling the risk of being bitten by malaria vectors in a vector control area in southern Benin, west Africa.** *Parasit Vectors* 2013, **6**:71.

66. Moss WJ, Hamapumbu H, Kobayashi T, Shields T, Kamanga A, Clennon J, Mharakurwa S, Thuma PE, Glass G: **Use of remote sensing to identify spatial risk factors for malaria in a region of declining transmission: a cross-sectional and longitudinal community survey.** *Malar J* 2011, **10**:163.

67. Musa MI, Shohaimi S, Hashim NR, Krishnarajah I: **A climate distribution model of malaria transmission in Sudan.** *Geospat Health* 2012, **7**:27–36.

68. Nath MJ, Bora A, Talukdar PK, Das NG, Dhiman S, Baruah I, Singh L: **A longitudinal study of malaria associated with deforestation in Sonitpur district of Assam, India**. *Geocarto Int* 2012, **27**:79–88.

69. Nihei N, Hashida Y, Kobayashi M, Ishii A: **Analysis of malaria endemic areas on the Indochina Peninsula using remote sensing.** *Jpn J Infect Dis* 2002, **55**:160–6.

70. Nobre A a., Schmidt AM, Lopes HF: **Spatio-temporal models for mapping the incidence of malaria in Para**. *Environmetrics* 2005, **16**:291–304.

71. Noor AM, Clements AC a, Gething PW, Moloney G, Borle M, Shewchuk T, Hay SI, Snow RW: **Spatial prediction of Plasmodium falciparum prevalence in Somalia.** *Malar J* 2008, **7**:159.

72. Noor AM, ElMardi K a, Abdelgader TM, Patil AP, Amine A a a, Bakhiet S, Mukhtar MM, Snow RW: **Malaria risk mapping for control in the republic of Sudan.** *Am J Trop Med Hyg* 2012, **87**:1012–21.

73. Noor AM, Gething PW, Alegana V a, Patil AP, Hay SI, Muchiri E, Juma E, Snow RW: **The risks of malaria infection in Kenya in 2009.** *BMC Infect Dis* 2009, **9**:180.

74. Noor AM, Uusiku P, Kamwi RN, Katokele S, Ntomwa B, Alegana V a, Snow RW: **The receptive versus current risks of Plasmodium falciparum transmission in Northern Namibia: implications for elimination.** *BMC Infect Dis* 2013, **13**:184.

75. Noor AM, Alegana VA, Patil AP, Moloney G, Borle M, Yusuf F, Amran J, Snow RW: **Mapping the receptivity of malaria risk to plan the future of control in Somalia.** *BMJ Open* 2012, **2**.

76. Oesterholt MJ a M, Bousema JT, Mwerinde OK, Harris C, Lushino P, Masokoto a, Mwerinde H, Mosha FW, Drakeley CJ: **Spatial and temporal variation in malaria transmission in a low endemicity area in northern Tanzania.** *Malar J* 2006, **5**:98.

77. Omukunda E, Githeko A, Ndong’a MF, Mushinzimana E, Yanr G: **Effect of swamp cultivation on distribution of anopheline larval habitats in Western Kenya**. *J Vector Borne Dis* 2013, **49**:61–71.

78. Omumbo J a, Hay SI, Goetz SJ, Snow RW, Rogers DJ: **Updating Historical Maps of Malaria Transmission Intensity in East Africa Using Remote Sensing.** *Photogramm Eng Remote Sensing* 2002, **68**:161–166.

79. Omumbo J a, Hay SI, Snow RW, Tatem a J, Rogers DJ: **Modelling malaria risk in East Africa at high-spatial resolution.** *Trop Med Int Health* 2005, **10**:557–66.

80. Peterson I, Borrell LN, El-Sadr W, Teklehaimanot A: **A temporal-spatial analysis of malaria transmission in Adama, Ethiopia.** *Am J Trop Med Hyg* 2009, **81**:944–9.

81. Qi Q, Guerra C a, Moyes CL, Elyazar IRF, Gething PW, Hay SI, Tatem AJ: **The effects of urbanization on global Plasmodium vivax malaria transmission.** *Malar J* 2012, **11**:403.

82. Rahman A, Krakauer N, Roytman L, Goldberg M, Kogan F: **Application of advanced very high resolution radiometer (AVHRR)-based vegetation health indices for estimation of malaria cases.** *Am J Trop Med Hyg* 2010, **82**:1004–9.

83. Raso G, Schur N, Utzinger J, Koudou BG, Tchicaya ES, Rohner F, N’goran EK, Silué KD, Matthys B, Assi S, Tanner M, Vounatsou P: **Mapping malaria risk among children in Côte d’Ivoire using Bayesian geo-statistical models.** *Malar J* 2012, **11**:160.

84. Reid H, Haque U, Clements AC a, Tatem AJ, Vallely A, Ahmed SM, Islam A, Haque R: **Mapping malaria risk in Bangladesh using Bayesian geostatistical models.** *Am J Trop Med Hyg* 2010, **83**:861–7.

85. Reid H, Vallely A, Taleo G, Tatem AJ, Kelly G, Riley I, Harris I, Henri I, Iamaher S, Clements AC a: **Baseline spatial distribution of malaria prior to an elimination programme in Vanuatu.** *Malar J* 2010, **9**:150.

86. Riedel N, Vounatsou P, Miller JM, Gosoniu L, Chizema-Kawesha E, Mukonka V, Steketee RW: **Geographical patterns and predictors of malaria risk in Zambia: Bayesian geostatistical modelling of the 2006 Zambia national malaria indicator survey (ZMIS).** *Malar J* 2010, **9**:37.

87. Rosa-Freitas MG, Tsouris P, Peterson a T, Honório NA, de Barros FSM, de Aguiar DB, Gurgel HDC, de Arruda ME, Vasconcelos SD, Luitgards-Moura JF: **An ecoregional classification for the state of Roraima, Brazil: the importance of landscape in malaria biology.** *Mem Inst Oswaldo Cruz* 2007, **102**:349–57.

88. Roux E, Gaborit P, Romaña C a, Girod R, Dessay N, Dusfour I: **Objective sampling design in a highly heterogeneous landscape - characterizing environmental determinants of malaria vector distribution in French Guiana, in the Amazonian region.** *BMC Ecol* 2013, **13**:45.

89. Salehi M, Mohammad K, Farahani MM, Zeraati H, Nourijelyani K, Zayeri F: **Spatial modeling of malaria incidence rates in Sistan and Baluchistan province, Islamic Republic of Iran**. *Saudi Med J* 2008, **29**:1791–1796.

90. Sinka ME, Bangs MJ, Manguin S, Coetzee M, Mbogo CM, Hemingway J, Patil AP, Temperley WH, Gething PW, Kabaria CW, Okara RM, Van Boeckel T, Godfray HCJ, Harbach RE, Hay SI: **The dominant Anopheles vectors of human malaria in Africa, Europe and the Middle East: occurrence data, distribution maps and bionomic précis.** *Parasit Vectors* 2010, **3**:117.

91. Sinka ME, Bangs MJ, Manguin S, Rubio-Palis Y, Chareonviriyaphap T, Coetzee M, Mbogo CM, Hemingway J, Patil AP, Temperley WH, Gething PW, Kabaria CW, Burkot TR, Harbach RE, Hay SI: **A global map of dominant malaria vectors.** *Parasit Vectors* 2012, **5**:69.

92. Sithiprasasna R, Ugsang DM, Honda K, Jones JW, Singhasivanon P, Sciences M, Applications ST, Thani P, Hygiene T: **Ikonos-derived malaria transmission risk in Northwestern Thailand**. *Southeast Asian J Trop Med Public Health* 2005, **36**:14–22.

93. Srivastava A, Nagpal BN, Saxena R, Wadhwa TC, Mohan S, Siroha GP, Prasad J, S. K. Subbarao: **Malaria epidemicity of Mewat region, District Gurgaon, Haryana, India: a GIS-based study**. *Curr Sci* 2004, **86**:1297–1303.

94. Srivastava A, Nagpal BN, Saxena R, Eapen A, Ravindran KJ, Subbarao SK, Rajamanikam C, Palanisamy M, Kalra NL, Appavoo NC: **GIS based malaria information management system for urban malaria scheme in India**. *Comput Methods Programs Biomed* 2003, **71**:63–75.

95. Staedke SG, Nottingham EW, Cox J, Kamya MR, Rosenthal PJ, Dorsey G: **Short report: proximity to mosquito breeding sites as a risk factor for clinical malaria episodes in an urban cohort of Ugandan children.** *Am J Trop Med Hyg* 2003, **69**:244–6.

96. Stefani A, Roux E, Fotsing J-M, Carme B: **Studying relationships between environment and malaria incidence in Camopi (French Guiana) through the objective selection of buffer-based landscape characterisations.** *Int J Health Geogr* 2011, **10**:65.

97. Stensgaard A-S, Vounatsou P, Onapa AW, Simonsen PE, Pedersen EM, Rahbek C, Kristensen TK: **Bayesian geostatistical modelling of malaria and lymphatic filariasis infections in Uganda: predictors of risk and geographical patterns of co-endemicity.** *Malar J* 2011, **10**:298.

98. Tatem AJ, Gething PW, Smith DL, Hay SI: **Urbanization and the global malaria recession Urbanization and the global malaria recession**. *Malar J* 2013, **12**.

99. Tatem AJ, Guerra C a, Kabaria CW, Noor AM, Hay SI: **Human population, urban settlement patterns and their impact on Plasmodium falciparum malaria endemicity.** *Malar J* 2008, **7**:218.

100. Temel T: **Malaria from the gap: need for cross-sector co-operation in Azerbaijan**. *Acta Trop* 2004, **89**:249–259.

101. Thomas CJ, Lindsay SW: **Local-scale variation in malaria infection amongst rural Gambian children estimated by satellite remote sensing.** *Trans R Soc Trop Med Hyg* 2000, **94**:159–63.

102. Townes LR, Mwandama D, Mathanga DP, Wilson ML: **Elevated dry-season malaria prevalence associated with fine-scale spatial patterns of environmental risk: a case-control study of children in rural Malawi.** *Malar J* 2013, **12**:407.

103. van der Hoek W, Konradsen F, Amerasinghe PH, Perera D, Piyaratne M, Amerasinghe FP: **Towards a risk map of malaria for Sri Lanka: the importance of house location relative to vector breeding sites**. *Int J Epidemiol* 2003, **32**:280–285.

104. Xiao D, Long Y, Wang S, Fang L, Xu D, Wang G, Li L, Cao W, Yan Y: **Spatiotemporal distribution of malaria and the association between its epidemic and climate factors in Hainan, China.** *Malar J* 2010, **9**:185.

105. Yang G, Gao Q, Zhou S, Malone JB, Mccarroll JC, Tanner M, Vounatsou P, Bergquist R, Utzinger J: **Mapping and predicting malaria transmission in the People’ s Republic of China , using integrated biology-driven and statistical models**. *Geospat Health* 2010, **5**:11–22.

106. Yang G-J, Tanner M, Utzinger J, Malone JB, Bergquist R, Chan EYY, Gao Q, Zhou X-N: **Malaria surveillance-response strategies in different transmission zones of the People’s Republic of China: preparing for climate change.** *Malar J* 2012, **11**:426.

107. Yang G, Zhou X, Malone JB, McCarroll JC, Wang T, Liu J: **Application of multifactor spatial composite model to predict transmission tendency of malaria at national level**. *Chinese J Parasitol Parasit Dis* 2002, **20**:145–147.

108. Yeshiwondim AK, Gopal S, Hailemariam AT, Dengela DO, Patel HP: **Spatial analysis of malaria incidence at the village level in areas with unstable transmission in Ethiopia.** *Int J Health Geogr* 2009, **8**:5.

109. Zacarias OP, Andersson M: **Mapping malaria incidence distribution that accounts for environmental factors in Maputo Province--Mozambique.** *Malar J* 2010, **9**:79.

110. Zacarias OP, Andersson M: **Spatial and temporal patterns of malaria incidence in Mozambique.** *Malar J* 2011, **10**:189.

111. Zayeri F, Salehi M, Pirhosseini H: **Geographical mapping and Bayesian spatial modeling of malaria incidence in Sistan and Baluchistan province, Iran.** *Asian Pac J Trop Med* 2011, **4**:985–92.

112. Zhou G, Sirichaisinthop J, Sattabongkot J, Jones J, Yan G, Cui L: **Spatio-temporal distribution of *Plasmodium falciparum* and *vivax* malaria in Thailand**. *Am J Trop Med Hyg* 2005, **72**:256–262.

113. Zhou S-S, Zhang S-S, Wang J-J, Zheng X, Huang F, Li W-D, Xu X, Zhang H-W: **Spatial correlation between malaria cases and water-bodies in Anopheles sinensis dominated areas of Huang-Huai plain, China.** *Parasit Vectors* 2012, **5**:106.
